# John Duane, Jr.

**Published:** 1895-12

**Authors:** 


JOHN DUANE, JR., D.V.S., one of New York City's veterinarians, a graduate of the American Veterinary College, Class of 1881, died recently in that city. As a student he was a genial and clever fellow among his classmates, and his extreme good nature made him many friends. Not a natural student by choice, after a few years of active practice in the lower part of New York City he followed the more natural bent of his nature, entering the domain of politics, where he was quickly accorded a lucrative place associated with the local judiciary of his city. He had little taste for association work, and was therefore but slightly known outside of his own city.

